# Tumor immune microenvironment (TIME) to enhance antitumor immunity

**DOI:** 10.1186/s40001-023-01125-3

**Published:** 2023-05-13

**Authors:** Sajin Rajbhandary, Hari Dhakal, Sudip Shrestha

**Affiliations:** 1 Department of Medical Oncology, Nepal Cancer Hospital and Research Center, Satdobato-Godawari Road, Lalitpur, Nepal; 2 Department of Laboratory Medicine and Pathology, Nepal Cancer Hospital and Research Center, Lalitpur, Nepal

**Keywords:** Tumor microenvironment, Tumor immune microenvironment, Immune hot tumors, Immune cold tumors, Anti-tumor immunity, Tumor immune infiltrates

## Abstract

The tumor microenvironment is a result of dynamic interaction between different cellular and non-cellular components. In its essence it is not a solo performer, but an ensemble of performers that includes cancer cells, fibroblasts, myo-fibroblasts, endothelial cells and immune cells. The short review highlights important immune infiltrates within the tumor microenvironment that shape cytotoxic t lymphocyte (CTL)-rich immune hot and CTL-deficient immune cold tumors and novel strategies that have potential role in enhancing our immune responses in both immune hot and immune cold tumors.

## Introduction

Tumors are identified by their cellular features that include cytoplasmic appearance, nuclear differentiation and atypical makeup. Thoroughly described by Hanahan et al., cancer cells are programmed to resist cell death, proliferate, evade growth suppressors, metastasize, induce angiogenesis, attain replicative immortality and reprogram metabolism. They have the potenital to avoid immune destruction, undergo non-mutational epigenetic programming and possess phenotypic plasticity [1]. The dynamics between these oncogenetic processes and intrinsic host response molds the environment around it. This tumor microenvironment (TME) consists of various cellular and non-cellular components which is effectively the result of intrinsic host response such as cytokines, chemokines, and inflammation responding to disrupting cancer behavior, tumor surface antigens and mechanisms of cell defense/ growth [2]. The entire avenue of disease prognosis and treatment response is affected by the understanding of these various components of the tumor microenvironment.

## Tumor immune microenvironment and immune infiltrates

One of the most significant components of the TME is the immune component. Immune infiltration and its role in cancer treatment were first reported more than a century back [3]. Immune infiltrates are the result of cancerous cells which are supported by two theories. First, cancer is antigenic and induces an active immune response [4] and second immune surveillance is vital for cancer progression and regression [5].

More recently, the concept of tumor immune microenvironment (TIME) has been well elaborated and their role in prognostication and treatment response has been established by various studies. A variety of immune cells including lymphocytic cells (T, B, natural killer cells), myeloid cells (neutrophils, macrophages, dendritic cells). Along with other effector and regulatory cells in adaptive immunity such as  Type 1T helper cells (Th1), Type 2T helper cells (Th2), T helper 17 cells (Th17), T regulatory cells (Treg) have been identified within the tumor microenvironment. Amongst these cells infiltrating lymphocytes, dendritic cells, t-follicular helper have favorable prognostic role whereas m2 macrophages, polymorph nuclear cells identified as non-favorable cellular types [6].

## Immune hot and immune cold tumors

Based on the characteristics of these immune infiltrates tumors can be classified as either immune hot or immune cold tumors. Immune hot tumors or t inflamed tumors usually express a higher number of programmed death ligand-1 (PDL-1) expressing tumors and infiltration of PD-1 expressing CTLs [7]. The infiltrating lymphoid and myeloid cells in the immune hot tumors have some unique functions such as, monocytes polarized into M1 types secrete lysosomes and destroy tumor cells. NK cells are capable of lysing cells with decreased expression of MHC proteins, e.g., virus infected cells, virus affected tumorous cells, mutating cancer cells [8]. Increased NK cell expression in tumor microenvironment (TME) milieu has shown better prognosis. Cytotoxic CD4 T cells (Th1, Th2 cells) not only provide immunologic help by enhancing anti-tumor cytotoxicity of CD8 T cells and macrophage activity, but also have direct action on cancer cells much like the CTLs which are both contact and granule dependent [9]. Most importantly, CTLs play a major role in our ability to recognize and destroy cancer cells, they do so via their ability to recognize tumor antigens presented on MHC I molecules and mount an effector function destroying malignant or pre malignant cells. Presence of CTL in tumor margins have proven good prognostic values in various cancers (e.g., colon cancer, lung cancer, melanoma [10–12] and its anti-tumor potential has been harnessed to develop effective immunotherapeutics drugs such as immune check point inhibitors and chimeric antigen receptor t cells (CARTs).

Immune cold tumors are t uninflamed tumors, they lack PDL-1 expressing tumor cells and PD-1 expressing CTL.A variety of reasons make some tumors immune cold, e.g., decreased antigenic expression [13] effective immune evasion methods [14], T cells repelling vasculature and high expression of immune suppressing immune infiltrates [15]. They have a characteristically increased expression of myeloid derived stromal cells (MDSC), M2 macrophages, PMNs, Tregs and Th17 cells [16, 17]. Tumor recruited MDSCs plays a central role in controlling and maintaining an immunosuppressive TME. It does so via immunologic and non-immunologic mechanism. MDSC scan remodel tissues with stromal components that are pro tumor, promote angiogenesis for tumor growth along with inhibition T cells proliferation, cytokine production and cytotoxic function [18]. In addition M2 polarized macrophages and T Regs support angiogenesis by secreting adrenomedullin and vascular epithelial growth factors (VEGFs) and express immunosuppressive molecules such as IL-10, programmed death-ligand 1 (PD-L1), and TGFβ, favoring tumor growth [19].

## Immunotherapeutic agents in immune hot and immune cold tumors

 Immune hot tumors have an endogenously activated T cell population. These are, however, suppressed by tumors expressing T cell inhibitory signals. Widely studied in clinical setting CTLA-4+/CD86, PD-1/PDL-1, MHC/LAG3 inhibitory pathway blockers have shown clinical benefit and is approved for treatment in a variety of advanced solid and hematologic malignancies [20, 21]. However, efficacy and durability of anti-tumor response with immune checkpoint inhibitors have a large space for improvement [22]. One of the key components for an effective and sustained immune response is APC activity. T cells need peptide-mhc complexes and co-stimulatory signals from APC to mount an effector response. These signals can be compounded with the use of CD40 agonists [23]. CD40 ligation on APCs induces increased surface expression of co-stimulatory and MHC molecules, production of pro-inflammatory cytokines, and enhanced T-cell triggering [24]. Hence CD40 agonists moAb can play an important role in enhancing APC function for a meaningful and sustained anti-tumor T cell function. Recently, immune check point refractory metastatic melanoma have demonstrated treatment benefit from combination use of CD40 agonistic antibody and nivolumab therapy [25]. Similarly, CD 40 moab (sotigalimab) had manageable toxicity and an early efficacy signal in a phase Ib pancreatic cancer trial [26].

Immune cold tumors are another side of the story it possesses a very unique and challenging clinical scenario. Besides being intratumorally CTL deficient [27], immune cold tumors contain effector T cells that have defective priming and homing signals. A deficient TAA, low MHC presentation of antigenic material, T cell/ DC interactions abnormal vasculature and immune suppressive TME, all contribute to defective priming/homing mechanisms in these types of tumor [28].  Thus treatment methods which could turn these unfavorable conditions to favorable ones are being explored. Among a few active agents CD137 agonist compounds in the form of monoclonal antibodies, CART cells and adoptive cell transfer have shown promising results. CD137 expressing T cells have been known to be highly cytotoxic. Moreover, in non-immunogenic tumors they can overcome effects of PDL1 inhibition and T reg associated suppression of microenvironment to mount an effective anti-tumor T cell activity [29]. In clinical setting CD137 agonist molecules (MCLA-145) in combination with PD-1 blocker are expected to enhance naïve T cell priming and promote long-term T cell immunity.

One of the major areas of interest in immune cold tumors is TAMS or M2 macrophages. They secrete IL-6, IL-8 promoting cancer cell growth and activate Th2 effector cells, and promote tumor angiogenesis via activation of VEGF and TGF beta. Furthermore they  release matrix metalloproteinases promoting metastasis. Besides modulating the microenvironment TAMS directly inhibit CD8T cell activity inducing the expression of PD-L1 in monocytes and secretion of numerous immunosuppressive cytokines and factors, including IL-10, TGF-β and ROS, leading to CD8+ TIL exhaustion and dysfunction. TAMs can also directly inhibit CD8+ T cells cytotoxicity through the depletion of the amino acids, such as l-arginine and tryptophan.

Therapies targeting TAMS are either cytotoxic to TAMS (trabectedin, bisphosphonates), prevent its migration and recruitment by tumors (anti-CSF-1/CSF-1R [30] CCL2/CCR2, or the CXCL12/CXCR4 axis [31] or reprogram them to anti-tumor M1 subtypes [32], or via anti-CD47 mabs [33]). Clinically, selective TAM inhibitor bemcentinib has shown not only improved PFS when used in combination with pembrolizumab, but also induced epithelial differentiation and returned the tumor microenvironment to an immune stimulatory phenotype [34].

## Conclusion

 Enhanced anti-tumor immunity in the form immune check point inhibitors has improved clinical outcomes, but a lot remains to be explored especially exploring novel methods to enhance anti-tumor functions and long-lived anti-tumor immune response. This review explores in brief a few novel ways of enhancing anti-tumor response by highlighting roles of some important immune cells in the tumor microenvironment and factors that can be considered in circumstances when current day immunologic armaments do not work. We are, however, unable to explore the world of long-lived tumor immunity and novel structures that could hold tumor specific clonal effector T cells. There is evidence suggesting naïve T cells are selectively in contact with mature DC in tumor-associated tertiary lymphoid structures (TLS) giving us some direction as TLS may be an active site for the priming and the proliferation but a lot remains to be explored. We conclude by highlighting the significant role of TME and its immune infiltrate in determining effective therapeutic strategies and opportunities to explore novel approach to cancer treatment in the coming future.

## Data Availability

Not applicable.
